# Natural Cyanobacterial Polymer-Based Coating as a Preventive Strategy to Avoid Catheter-Associated Urinary Tract Infections

**DOI:** 10.3390/md18060279

**Published:** 2020-05-26

**Authors:** Bruna Costa, Rita Mota, Paula Tamagnini, M. Cristina L. Martins, Fabíola Costa

**Affiliations:** 1 i3S—Instituto de Investigação e Inovação em Saúde, Universidade do Porto, Rua Alfredo Allen, 208, 4200-135 Porto, Portugal; bruna.costa@i3s.up.pt (B.C.); rita.mota@ibmc.up.pt (R.M.); pmtamagn@ibmc.up.pt (P.T.); cmartins@ineb.up.pt (M.C.L.M.); 2 INEB—Instituto de Engenharia Biomédica, Universidade do Porto, Rua Alfredo Allen, 208, 4200-135 Porto, Portugal; 3 IBMC—Instituto de Biologia Molecular e Celular, Universidade do Porto, Rua Alfredo Allen, 208, 4200-135 Porto, Portugal; 4 Faculdade de Ciências, Departamento de Biologia, Universidade do Porto, Rua do Campo Alegre, Edifício FC4, 4169-007 Porto, Portugal; 5 ICBAS—Instituto de Ciências Biomédicas Abel Salazar, Universidade do Porto, Rua Jorge de Viterbo Ferreira 228, 4050-313 Porto, Portugal

**Keywords:** cyanobacteria, uropathogens, anti-adhesive coating, urinary catheters, surface modification, catheter-associated urinary tract infections

## Abstract

Catheter-associated urinary tract infections (CAUTIs) represent about 40% of all healthcare-associated infections. Herein, the authors report the further development of an infection preventive anti-adhesive coating (CyanoCoating) meant for urinary catheters, and based on a natural polymer released by a marine cyanobacterium. CyanoCoating performance was assessed against relevant CAUTI etiological agents, namely *Escherichia coli*, *Proteus mirabilis*, *Klebsiella pneumoniae,* methicillin resistant *Staphylococcus aureus* (MRSA), and *Candida albicans* in the presence of culture medium or artificial urine, and under biofilm promoting settings. CyanoCoating displayed a broad anti-adhesive efficiency against all the uropathogens tested (68–95%), even in the presence of artificial urine (58–100%) with exception of *P. mirabilis* in the latter condition. Under biofilm-promoting settings, CyanoCoating reduced biofilm formation by *E. coli*, *P. mirabilis,* and *C. albicans* (30–60%). In addition, CyanoCoating prevented large crystals encrustation, and its sterilization with ethylene oxide did not impact the coating stability. Therefore, CyanoCoating constitutes a step forward for the implementation of antibiotic-free alternative strategies to fight CAUTIs.

## 1. Introduction

Urinary catheters are the most common indwelling device, with 15–25% of hospitalized patients undergoing catheterization [1]. More than 30 million urinary catheters are used per year to manage urinary incontinence and urinary retention, during and/or after surgical practices in the USA only [2]. Infection is the main concern associated with the use of catheters (either long- or short-term). Catheter-associated urinary tract infections (CAUTIs) account for approximately 40% of all healthcare-associated infections; therefore, are associated to major economic burden ($1000 per treatment of CAUTI in USA) [3]. This problem is rising together with bacterial antibiotic resistance, which is considered by the World Health Organization (WHO) as one of the most severe health threats around the world [4]. CAUTI establishment is related with the impairment of the natural defense systems of the healthy urological mucosa. When the use of a catheter is required, the natural flush of bacteria by micturition is hampered [5]. Moreover, damage to the inner walls of the urinary system breaches the natural protection against bacterial adhesion, which, adding to the presence of a foreign material and a compromised immune system, contributes to the establishment of CAUTIs. CAUTIs arise from cross contamination derived from the patient’s normal fecal flora or from the healthcare personnel handling [6]. These infections are always associated with the occurrence of microbial biofilms, being the most prevalent Gram-negative bacteria, such as *Escherichia coli*, *Klebsiella pneumoniae*, *Proteus mirabilis,* and *Pseudomonas aeruginosa*, Gram-positive *Staphylococcus aureus* (including methicillin-resistant strains), and yeasts—particularly *Candida* species, etiological agents that are particularly well adapted to the urinary tract microenvironment [7].

CAUTIs are a major cause of catheter encrustation, which is promoted by urease-positive pathogens, such as *P. mirabilis*, *P. aeruginosa,* and *K. pneumoniae* [8]. Urease catalyzes the hydrolysis of urea into ammonia and carbamate, which in turn increases the urine pH promoting the formation of crystals [9]. The formation of biofilm itself may also promote catheter occlusion by the large amount of mucoid material produced (e.g., by *P. aeruginosa, K. pneumoniae*) or by the emergence of hyphae (e.g., *C. albicans*). Other CAUTI associated complications include bladder stones, septicemia, endotoxic shock, and pyelonephritis contributing to patients’ suffering, and frequently worsening other concomitant chronic pathologies [10]. In this way, new strategies are needed to optimize patient safety, control costs, and to reduce bacterial resistance. The current materials used to produce catheters include polyurethanes (PUs), silicone, polytetrafluoroethylene (PTFE), polyvinylchloride (PVC), and latex rubber [8]. PUs are among the best choices for biomedical applications due to their mechanical properties, namely durability, elasticity, fatigue resistance, and compliance [8]. The advantage of using PUs instead of silicone for urinary catheters is that PUs originate catheters with larger internal diameters (due to thinner walls) that are less prone to occlusion, and soften within the patient’s body, becoming more comfortable [8,11]. 

The most promising approach to improve urinary catheter safety is to alter its surface to avoid biofilm formation preventing the consequent infection [12,13,14,15]. For the development of anti-adhesive surfaces, natural polymers, such as hyaluronic acid and heparin, can be used [15,16,17]. Polysaccharides from marine sources, such as alginate, ulvan, agarose, and carrageenans have also been reported as possible alternatives [16,18,19]. Previously, Costa et al. [20] developed CyanoCoating, a coating based on a well-characterized extracellular polymer produced by a marine cyanobacterium [21]. These authors demonstrated that CyanoCoating has anti-adhesive properties against *S. aureus*, *S. epidermidis*, *P. aeruginosa*, and *E. coli*, and is biocompatible, having the potential to be applied to a wide range of medical devices, including blood contacting materials [20]. 

The present study is aimed at evaluating CyanoCoating capability to endure urinary catheter specifications (urine, uropathogens, and sterilization). Moreover, the absence of contaminants in the raw biological material was confirmed. Overall, the results obtained highlight the translational potential of CyanoCoating to mitigate challenges imposed by CAUTIs. 

## 2. Results

### 2.1. Biopolymer Regulatory Compliance Assessment: Metal and Microbial Contamination

The extracellular cyanobacterial polymer, mainly of heteropolysaccharidic nature, used to prepare the CyanoCoating is a new material not yet described on pharmacopeia, thus, metal and microbial contamination was addressed. The Inductively Coupled Plasma–Atomic Emission Spectrometry (ICP–AES) results showed that the isolated biopolymer was not contaminated with arsenic (As), cadmium (Cd), lead (Pb), or mercury (Hg) (Supplementary Table S1). Moreover, the microbiological assays showed that the biopolymer was not contaminated with bacteria or fungi, even before the autoclave sterilization process, as no colony-forming units (CFUs) where observed up to 5 days. 

### 2.2. CyanoCoating Surface Characterization

**Figure 1:**
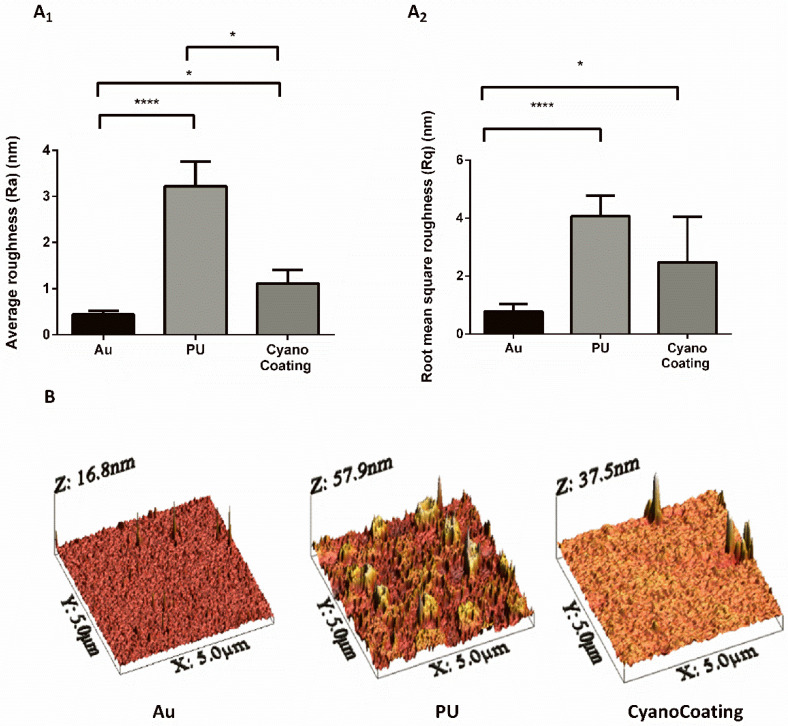
Characterization, by atomic force microscopy (AFM), of the surface roughness of gold substrates (Au), Au substrates coated with a polydopamine (pDA) layer plus polyurethane (PU), or Au substrates coated with a pDA layer plus CyanoCoating. (**A_1_**) Average roughness (Ra), (**A_2_**) Root mean square roughness (Rq) and (**B**) AFM three-dimensional (3D) surface images. Statistical analysis was performed by non-parametric Kruskal–Wallis analysis and statistical differences are indicated with * (*p* < 0.05) and **** (*p* < 0.001).

CyanoCoating was previously characterized in terms of thickness and wettability [20]. However, since surface topography is known to impact biofilm development, this parameter was evaluated by atomic force microscopy (AFM). CyanoCoating and medical grade polyurethane (PU) were covalently bound through a polydopamine (pDA) layer to gold (Au) substrates, as previously described [20]. The deposition of either the pDA + CyanoCoating or the control pDA + PU increased significantly surface roughness of Au substrates, as depicted in Figure 1A. CyanoCoating exhibited a smoother surface in comparison with PU, as demonstrated by the decrease of the average roughness (Ra) (Figure 1A_1_) and the root mean square roughness (Rq) (Figure 1A_2_). Representative AFM three-dimensional (3D) images of the threes surfaces can be observed in Figure 1B.

### 2.3. CyanoCoating Biological Performance

#### 2.3.1. Microbial Adhesion Assays

**Figure 2:**
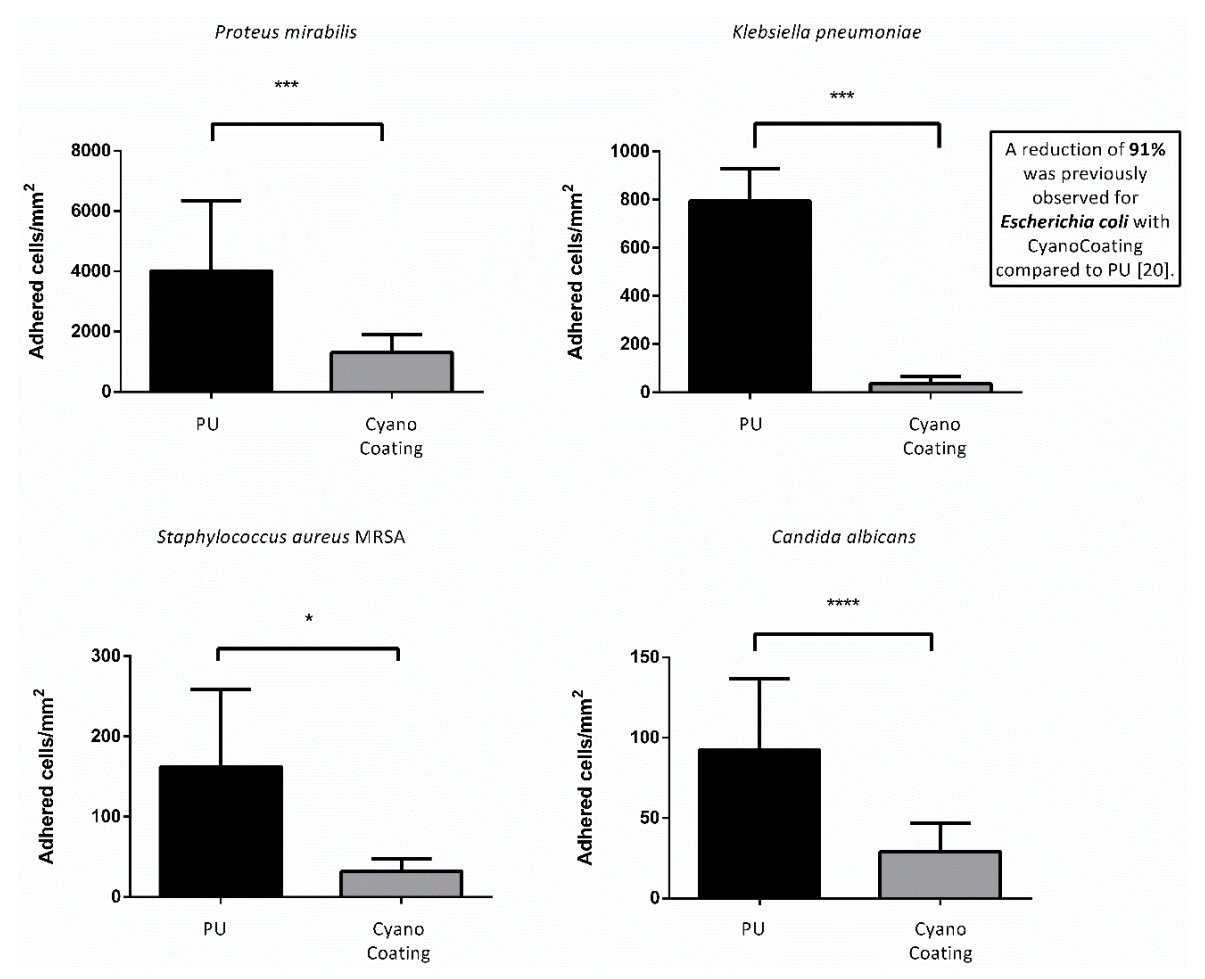
CyanoCoating anti-adhesive performance compared to medical grade polyurethane (PU). The coatings were tested against the uropathogens mentioned above each graph using the respective growth medium; see Materials and Methods. Data represent mean ± Standard deviation (n = 9). The assay was performed according to ISO 22196. Statistical analysis was performed by non-parametric Kruskal–Wallis analysis and statistical differences are indicated with * (*p* < 0.05), *** (*p* < 0.005) and **** (*p* < 0.001).

As the anti-adhesive performance of CyanoCoating was previously evaluated against *Escherichia coli* and *Pseudomonas aeruginosa* [20], herein, we focused on other relevant uropathogens for catheter-associated urinary tract infections (CAUTIs): *Proteus mirabilis*, *Klebsiella pneumoniae*, methicillin resistant *Staphylococcus aureus* (MRSA), and *Candida albicans* [22], according to ISO 22196:2007 [23]. Overall, the results obtained (Figure 2 and Figure S1) showed that microbial adhesion to CyanoCoating was significantly lower than the adhesion to medical grade PU, ranging from 68 ± 28% (*P. mirabilis*) to 95 ± 48% (*K. pneumoniae*). Importantly, CyanoCoating was efficient in preventing *S. aureus* (MRSA) adhesion (80 ± 27%), a microorganism that is very difficult to eradicate. Moreover, CyanoCoating could also reduce in 69 ± 19% the adhesion of the yeast *C. albicans* (responsible for 10–15% of CAUTIs [24]).

#### 2.3.2. Microbial Adhesion Assays with Artificial Urine

**Figure 3:**
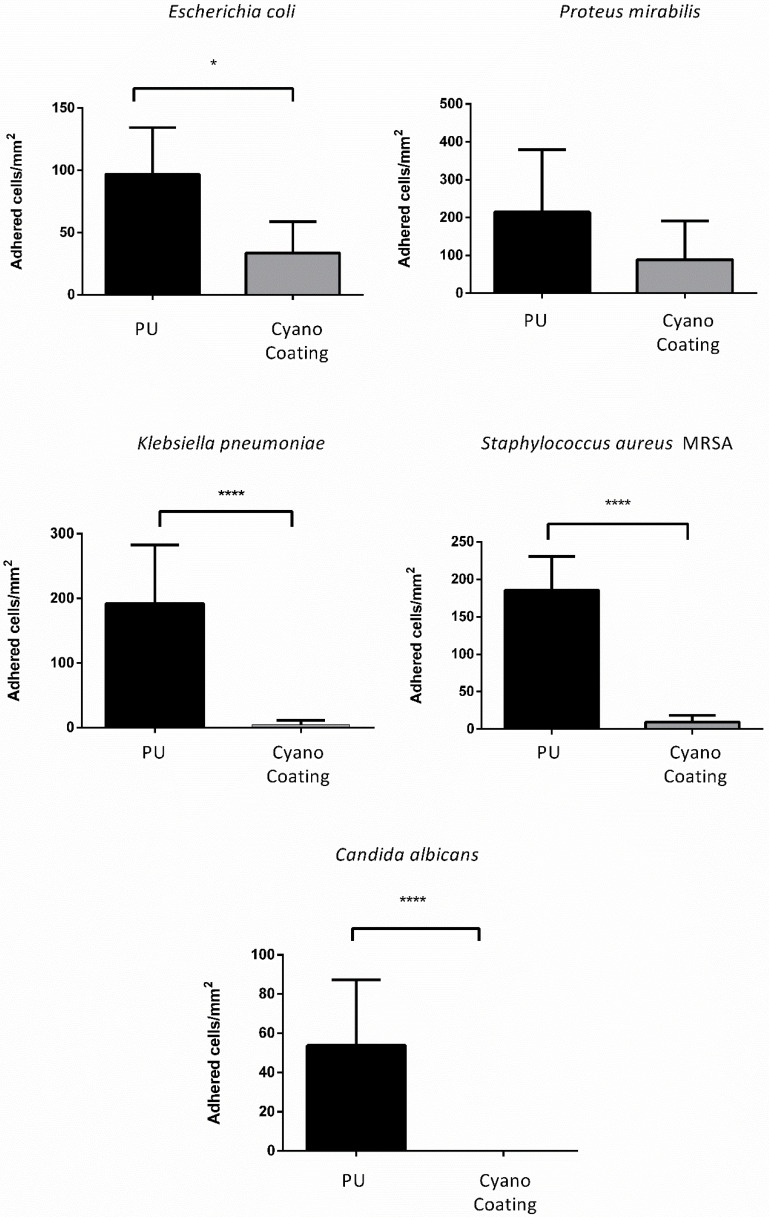
CyanoCoating anti-adhesive performance compared to medical grade polyurethane (PU) with artificial urine medium. The coating was tested against the uropathogens mentioned above each graph. Data represent mean ± Standard deviation (n = 9). The assay was performed according to ISO 22196. Statistical analysis was performed by non-parametric Kruskal–Wallis analysis and statistical differences are indicated with * (*p* < 0.05) and **** (*p* < 0.001).

The anti-adhesive performance of CyanoCoating was subsequently assessed with artificial urine medium (AUM) against *E. coli*, *P. mirabilis, K. pneumoniae, S. aureus* (MRSA) and *C. albicans*, also according to ISO 22196:2007. In the presence of AUM, CyanoCoating significantly reduced the adhesion of most of the uropathogens compared to PU. For the Gram-negative *E. coli* and *K. pneumoniae* a reduction of 65 ± 28% and 98 ± 54%, respectively, was observed, while for the Gram-positive *S. aureus* (MRSA) and the yeast *C. albicans*, a striking 95 ± 34% and 100% reduction, respectively, was observed (Figure 3 and Figure S2). 

#### 2.3.3. Biofilm Formation

**Figure 4:**
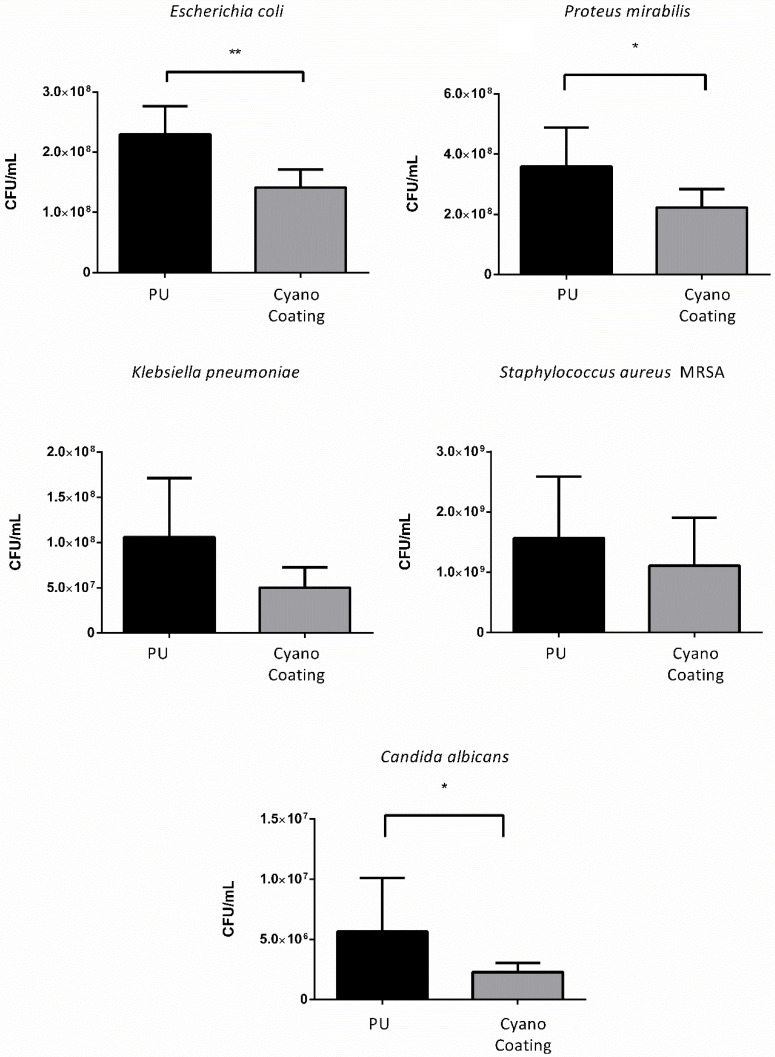
Effect of CyanoCoating on the prevention of biofilm formation compared to medical grade polyurethane (PU), by measuring the bacteria detached from the surfaces. Data represent mean ± Standard deviation (n = 9). Statistical analysis was performed by Mann–Whitney test (*t*-test) analysis and statistical differences are indicated with * (*p* < 0.05) and ** (*p* < 0.01).

In order to evaluate CyanoCoating effectiveness in preventing biofilm formation, a biofilm assay was performed according to Costa et al., [25]. After 24 h, the number of CFUs detached from the surfaces by sonication were determined. The efficiency of the sonication process was verified by observing the surfaces using inverted fluorescence microscopy. A reduction trend on biofilm formation was observed for *E. coli* (39 ± 10%), *P. mirabilis* (39 ± 15%) and *C. albicans* (60 ± 30%) on CyanoCoating samples compared to the control PU, while for *K. pneumoniae* and *S. aureus* MRSA no significant differences were observed (Figure 4). 

### 2.4. Encrustation Development 

**Figure 5:**
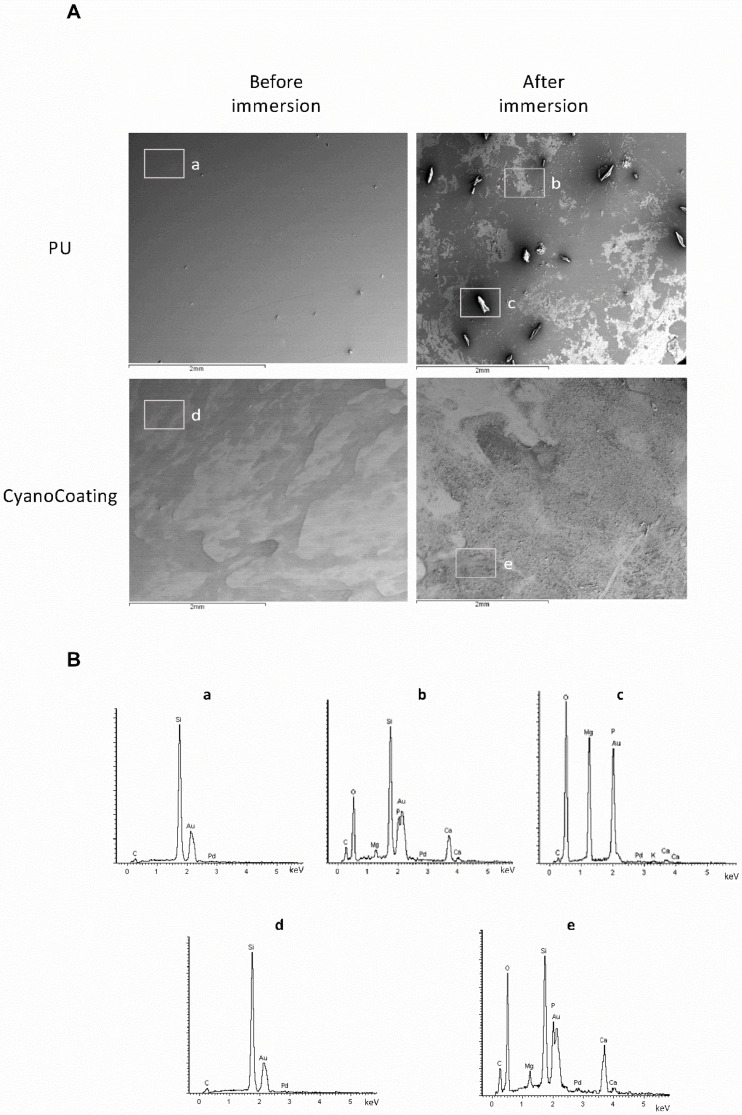
Encrustation development on CyanoCoating compared to medical grade polyurethane (PU). (**A**) SEM micrographs of the coatings before and after 7 days immersion in supplemented artificial urine medium (AUS). Magnification 30×. (**B**) Energy-dispersive X-ray spectroscopy (EDS) spectra of the selected areas on each coating surfaces before and after 7 days of immersion in AUS. **a** to **e** correspond to the areas highlighted in the SEM micrographs above.

Salts deposition on top of CyanoCoating, was evaluated by scanning electron microscopy (SEM) and energy-dispersive X-ray spectroscopy (EDS), after incubation with supplemented artificial urine medium (AUS). This urine is supplemented with urease and ovalbumine to promote an encrustation environment [26,27]. SEM micrographs (Figure 5A, left panel) show the clean surfaces of the CyanoCoating and the control PU at the initial time points (before the immersion in AUS). Seven days after the immersion, it was possible to observe salt deposition on top of the samples (Figure 5A, right panel), particularly in the PU surface where agglomerates of larger crystals are clearly visible. The EDS spectra (Figure 5B) indicate the presence of elements that could suggest the formation of struvite (NH_4_MgPO_4_·6H_2_O), brushite (CaHPO_4_·2H_2_O), or hydroxyapatite (Ca_5_(PO_4_)_3_(OH)) in the surface of the samples immersed in AUS (Figure 5B, lower panel). Higher amounts of magnesium (Mg) and phosphorus (P) were found on PU samples suggesting accumulation of struvite, whereas the presence of calcium (Ca) and P on CyanoCoating suggests accumulation of brushite or hydroxyapatite. Before immersion in AUS, EDS spectra of PU and CyanoCoating samples only present silicon (Si), gold (Au) and carbon (C), the expected elements of the substrates, and palladium (Pd) from the SEM analysis sputtering. 

### 2.5. CyanoCoating Stability After Sterilization

**Figure 6:**
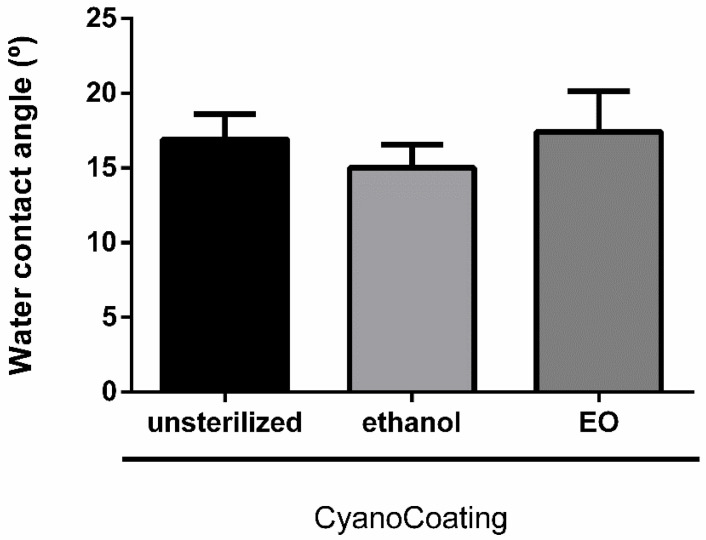
Surface characterization, by water contact angle (captive bubble method), of CyanoCoating samples without sterilization, after sterilization with ethanol 70% (*v*/*v*) or with ethylene oxide (EO). Data represent mean ± Standard deviation (n = 9).

**Figure 7:**
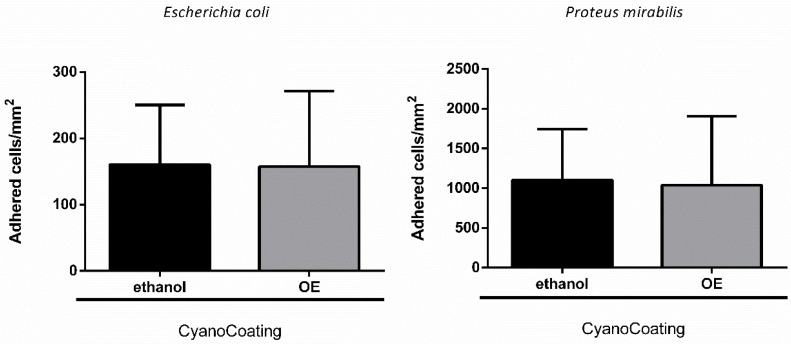
CyanoCoating anti-adhesive performance after sterilization with ethanol 70% (*v*/*v*) or ethylene oxide (EO). Data represent mean ± Standard deviation (n = 9). Adhesion of *Escherichia coli* and *Proteus mirabilis* in Tryptic Soy Broth (TSB). The assay was performed according to ISO 22196.

To evaluate the stability of CyanoCoating after sterilization by ethylene oxide (EO), the most common industrial sterilization technique for medical devices [28] and compatible with most of the biomaterials used in their manufacture, samples were characterized both physically (water contact angle measurements) and biologically (anti-adhesive performance). Our results revealed that EO sterilization did not significantly alter CyanoCoating wettability, compared to unsterilized samples, and samples submitted to the regular laboratorial ethanol-based disinfection protocol (Figure 6). Similarly, the anti-adhesive performance of CyanoCoating after EO sterilization was not altered, compared to samples submitted to the ethanol-based disinfection protocol, using *E. coli* or *P. mirabilis* as model bacteria (Figure 7).

## 3. Discussion

Among all healthcare-associated infections, catheter-associated urinary tract infections (CAUTIs) are recognized as the most prevalent worldwide [29]. In this work, we explore the possibility of a previously developed anti-adhesive coating, CyanoCoating [20], to endure urinary catheters specifications.

Concerning the quality of the raw material (cyanobacterial extracellular polymer) used to produce CyanoCoating, the absence of fungi and bacteria indicate that all steps performed from the cell cultures to the polymer extraction ensured a high purity level of the product, fulfilling the quality requirements suggested by pharmacopeia, and the regulations imposed by healthcare authorities. 

In the previous work, the broad-spectrum activity of CyanoCoating was assessed against relevant etiological agents responsible for medical devices-associated infections, including the uropathogens *Escherichia coli* and *Pseudomonas aeruginosa* (reducing bacterial adhesion by at least 80%) [20]. Here, the potential of CyanoCoating for CAUTIs mitigation was assessed against other relevant uropathogens, namely *Proteus mirabilis*, *Klebsiella pneumoniae*, methicillin-resistant *Staphylococcus aureus* (MRSA), and the yeast *Candida albicans* [30,31,32]. Overall, CyanoCoating greatly impaired the adhesion of the tested microorganisms (ranging from 68 ± 28% to 95 ± 48%). Considering that CyanoCoating is highly hydrophilic and exhibits a smoother topography compared to polyurethane (as visible in the AFM images) the hypothesis of an anti-adhesive mechanism of action is the most plausible. It is known that highly hydrophilic surfaces prevent the adsorption of proteins/cells due to the establishment of a hydration layer formed by well-structured water molecules linked to the surface by hydrogen bonds that works as a physical barrier [33]. In addition, the lack of bactericidal activity previously reported [20] reinforce our hypothesis. Similar results were obtained by other authors, using poly(ethylene glycol) (PEG) [34] or sulfobetaine methacrylate (SBMA) [35] anti-adhesive synthetic coatings onto PU or silicone surfaces, with *E. coli* and *S. epidermidis* or *P. aeruginosa* and *S. aureus* only. Our results demonstrate that CyanoCoating is effective against a broader range of microorganisms, including urease-positive bacteria and yeasts (this work and [20]). 

To better mimic the in vivo environment that bacteria encounter in the urinary tract [32,36], artificial urine medium was used for the in vitro adhesion assays. The microorganisms were chosen since *E. coli* is the most prevalent in CAUTIs, *P. mirabilis* is responsible for the most severe cases, *C. albicans* causes 10–15% of these infections and the other bacteria are also relevant [22,37]. In the presence of artificial urine medium, the overall microbial adhesion to CyanoCoating and PU surfaces was significantly lower than with culture medium, in particular for the Gram-negative bacteria *E. coli*, *P. mirabilis,* and *K. pneumoniae*. This result can be associated to the media composition; culture media promote bacterial growth and biofilm formation mechanisms since they contain glucose as a carbon source, in contrast with the artificial urine medium. Nevertheless, CyanoCoating performance was much better than PU against all the microorganisms tested, in particular for *K. pneumoniae* and *C. albicans*. In addition, we demonstrated that the efficiency of CyanoCoating was not negatively affected by clinically relevant sterilization procedures such as ethylene oxide (EO).

The efficiency of CyanoCoating on preventing biofilm formation was assessed against all uropathogens mentioned above. This method counts the CFUs originated after detachment of the biofilm by sonication instead of other indirect methods commonly used, e.g. the resazurin assay that assess the metabolic activity of bacteria in biofilms [38] or the canonical crystal violet assay that stains the extracellular matrix [38]. This last method cannot be used here due to the heteropolysaccharidic nature of the polymer used to generate the CyanoCoating [21]. Biofilm formation was significantly impaired for *E. coli*, *P. mirabilis,* and *C. albicans* ranging from 39 ± 10% to 60 ± 30%, suggesting that CyanoCoating hinders biofilm formation against a broad-spectrum of microorganisms, even for the difficult to eradicate fungi *C. albicans* [39]. Our results reinforce the strategy of using natural polymers to prevent biofilm formation, as reported by others, e.g., the use of carboxymethyl chitosan to coat medical grade silicone and that reduced biofilm formation by Gram-negative bacteria [37], or the low-molecular weight chitosan hydrogels used to coat polystyrene microplates that avoid biofilm formation by *Candida* spp. [39]. Current technologies in the market are based on the release of antimicrobial agents by the coating, such as antiseptics or antibiotics, to inhibit the colonization of the catheters. However, in spite of the broad-spectrum activity, these coatings exhaust their antimicrobial activities over long periods, are associated with toxicity and contribute for the development of antimicrobial resistance [2,40]. Having in mind the goal of developing an antibiotic-free coating, CyanoCoating may be combined with bactericidal compounds, such as antimicrobial peptides, that can be either immobilized or delivered [41,42]. 

Another critical aspect on indwelling urinary catheters is the mineral deposition on their surfaces. Frequently urinary catheters become blocked by hard mineral deposits, resulting in urine leakage, discomfort to the patient, and even catheter encrustation. In the worst-case scenario, the encrustation can only be solved by removing the catheter, which may cause trauma to the urethra [26,43]. The encrustation is exacerbated by the presence of urease positive pathogens, such as *P. mirabilis* [26]. Therefore, we challenge CyanoCoating with artificial urine medium supplemented with urease, which also contains albumin that mimics the bacterial and cellular debris that infected urine frequently contains [26]. The energy-dispersive X-ray spectroscopy (EDS) results clearly indicated the presence of Ca, P, Mg, and O that could suggest struvite (NH_4_MgPO_4_·6H_2_O), brushite (CaHPO_4_·2H_2_O) or hydroxyapatite (Ca_5_(PO_4_)_3_(OH)) formation. However, while on the control PU surface big rectangular shaped crystals protruded from the surface suggesting the formation of struvite [44], on the CyanoCoating individual crystallites with powdery appearance and smaller in size were formed, which is consistent with brushite or hydroxyapatite [45,46]. All together, these results show that CyanoCoating is less prone to encrustation, and therefore less prone to promote catheter blockage. 

## 4. Materials and Methods

### 4.1. Cyanobacterium Growth Conditions and Biopolymer Isolation 

The unicellular cyanobacterium *Crocosphaera chwakensis* CCY0110 [47] (previously identified as *Cyanothece* sp. CCY 0110; Culture Collection of Yerseke, The Netherlands; kindly provided by Lucas Stal) was grown in 2 L bioreactors with ASNIII medium, at conditions previously described [20,21]. Cells were grown until an optical density at 730 nm of approximately 2.5–3.5 and the extracellular biopolymer was isolated as previously described [20]. 

### 4.2. Biopolymer Contaminants 

#### 4.2.1. Assessment of Metal Contaminants

To assess the putative contamination of the cyanobacterial polymer with heavy metals, the presence of arsenic (As), cadmium (Cd), lead (Pb), and mercury (Hg) was evaluated. For this purpose, aqueous polymer solutions 0.5% (*w*/*v*) were prepared and mineralized using 5% HNO_3_ (*v*/*v*). Then, an Inductively Coupled Plasma–Atomic Emission Spectrometer (ICP–AES) (Ultima, Jobin Yvon), equipped with a 40.68 MHz RF generator and a Czerny-Turner monochromator with 1.00 m) was used for metals quantification. 

#### 4.2.2. Polymer Microbiological Control 

To assess the microbiological quality of the raw material, polymer bioburden (contamination with bacteria or fungi) was evaluated by microbiological assays as recommended by Portuguese Pharmacopeia [48]. To perform the assays, 10 mL of polymer solution 1% (*w*/*v*) were filtered by a 0.45 μm filter (Merck). Then, the filter was cut into halves and each part was placed on top of either Tryptic Soy Agar (TSA) plates or Sabouraud Dextrose Agar (SDA). After 24 h incubation period at 37 °C, the number of colonies-forming units (CFUs) were counted. Two replicates of each condition were performed.

### 4.3. CyanoCoating Development 

CyanoCoating was prepared as previously reported by [20]. Briefly, gold substrates (Au) were cleaned for 5 min, with ‘‘piranha’’ solution (7 parts of sulfuric acid (95%, *v*/*v* (BDH Prolabo): 3 parts of hydrogen peroxide (Merck), (CAUTION: this solution reacts violently with organic solvents and should be handled with care). Then, substrates were immersed in freshly prepared dopamine solution (2-(3,4-Dihydroxyphenyl)ethylamine hydrochloride, Sigma-Aldrich) (2 mg/mL in 10 mM TrisHCl pH 8.5) to allow formation of a polydopamine (pDA) layer on top of the Au substrate [20]. Subsequently, the polymer solution at 0.5% (*w*/*v*) was spin-coated (model WS-650-23, Laurell Technologies Corporation, North Wales, PA, USA) at 9000 rpm for 1 min on top of pDA-coated Au substrate. As control samples, medical grade polyurethane (PU) surfaces were prepared by similarly spin-coating the PU (Pellethane 2363 80 AE; Velox) solution at 0.1% (*w*/*v*) in tetrahydrofuran (Merck), on top of pDA-coated Au substrate [20]. 

### 4.4. CyanoCoating Surface Characterization by Atomic Force Microscopy (AFM)

Atomic Force Microscopy (AFM) images were obtained using a PicoPlus 5500 controller (Keysight Technologies, Santa Rosa, CA, USA). The images of gold substrate were performed in Tapping Mode, in air using a bar-shaped cantilever with a spring constant (k) in the range of 1–5 N/m (AppNano, Mountain View, CA, USA). The images on polyurethane (PU) and CyanoCoating were obtained in Contact Mode, in air, using a triangular shape cantilever V-shaped cantilever with a spring constant k = 0.085 N/m (Hydra-All-G, AppNano, Mountain View, CA, USA). The scan speed was set at 1.0 l/s, for both AFM modes. The scan size was 5 × 5 μm^2^. The software used to obtain the images was the PicoView 1.2 (Keysight Technologies, Santa Rosa, CA, USA). The WSxM5.0 software (Nanotec Electronica, Feldkirchen, Germany) was used to perform the roughness surface measurements [49]. 

### 4.5. CyanoCoating Biological Performance Evaluation

#### 4.5.1. Microbial Strains, Media, and Growth Conditions 

*P. mirabilis* (clinical isolate provided by Faculdade de Medicina Dentária, Universidade do Porto) was grown on cystine-lactose-lectrolyte-deficient agar (CLED agar) (Merck) and tryptic soya broth (TSB) (Merck). *E. coli* (ATCC 25922) and *S. aureus* MRSA (ATCC 33591), obtained from the American Type Culture Collection (ATCC), were grown on tryptic soya agar (TSA) (Merck) and TSB (Merck). *K. pneumoniae* (clinical isolate provided by Centro Hospitalar do Porto) was grown on TSA and Todd Hewitt Broth (THB). *C. albicans* (DSM 1386), obtained from the German Collection of Microorganisms and Cell Cultures GmbH (DSM), was grown on Sabouraud Dextrose Agar (SDA) (Merck) and Sabouraud Dextrose Broth (SDB) (Merck). The initial microbial inoculum was adjusted in TSB for *E. coli* and *S. aureus*, in THB for *K. pneumoniae*, or in SDB for *C. albicans*, according to OD_600nm_ measurement and subsequently confirmed by count of CFUs. 

#### 4.5.2. Microbial Adhesion Assays

Microbial adhesion assays were performed using *P. mirabilis*, *K. pneumoniae*, *S. aureus* MRSA and *C. albicans* according to ISO 22196:2007 (Plastics—Measurement of antibacterial activity on plastics surfaces) [23]. For CyanoCoating and PU disinfection, samples were immersed subsequently for 15 min, twice in ethanol 70% (Merck) and twice in filtered type II water (0.22 µm syringe filter), being dried with argon stream in a flow hood, and then transferred to a 24-well plate. Then, a 5 µL inoculum drop (1.8 × 10^6^ CFUs/mL) was placed on top of the samples and then covered with a previously sterilized polypropylene (PP) coverslip (Ø 9 mm), using the method described above. Samples were incubated for 24 h at 37 °C in moisturized condition. After 24 h, samples were rinsed with Phosphate Buffered Saline (PBS) three times. Adhered bacteria or fungi were fixed with paraformaldehyde 4% (*v*/*v*) in PBS, for 30 min at room temperature (RT). After rinsing with PBS three times, samples were stained with 4′,6-diamidino-2-phenylindole (DAPI) (0.1 μg/mL) for 30 min at RT, protected from light. Afterwards, samples were rinsed with PBS and transferred to an uncoated 24-well μ-plate (#82406, IBIDI, Gräfelfing, Germany) with the surface facing the bottom. Results represent average of three independent assays, with three replicates per sample.

High-content screening microscope (IN Cell Analyzer 2000, GE Healthcare, Chicago, IL, USA) with a Nikon 20× / 0.95 NA Plan Apo objective (binning 1 × 1), using a charge-coupled device (CCD) Camera (CoolSNAP K4) was used to observe samples from microbial adhesion assays. Image field of view (FOV) x-y for this objective is 0.8 × 0.8 cm. Moreover, 9 FOV per sample were acquired spanning an area of 5.76 cm^2^. The excitation and emission filters used were DAPI (excitation: 365 nm; emission: 420 nm). On-the-fly deconvolution was performed. The number of adherent bacteria were quantified using the ImageJ software, and values were converted to bacteria per mm^2^. 

Adhesion reduction percentages were calculated according to the formula: [number of adhered bacteria per mm^2^ on CyanoCoating × 100]/[number of adhered bacteria per mm^2^ on PU]. The standard deviations were calculated considering error propagation of the measurements uncertainties.

#### 4.5.3. Antimicrobial Adhesion Assays in the Presence of Artificial Urine Medium

To better simulate the conditions of microbial adhesion inside urinary tract, the anti-adhesive performance of CyanoCoating against *E. coli, P. mirabilis, K. pneumoniae, S. aureus MRSA,* and *C. albicans* was performed as explained previously (see Section 4.5.2.), but using artificial urine medium prepared according to Brooks et al. [32] (composition: Supplementary Table S2) to adjust initial inoculum. After 24 h incubation period, samples were processed, as described in Section 4.5.2., the number of adherent bacteria were quantified using the ImageJ software, and values were converted to bacteria per mm^2^. Results represent average of three independent assays, with three replicates per sample. The adhesion reduction percentages and respective standard deviations were calculated, as described in Section 4.5.2.

#### 4.5.4. Biofilm Formation Assessment

*E. coli, P. mirabilis, K. pneumoniae, S. aureus* MRSA, and *C. albicans* were grown overnight in respective culture media, described in Section 4.5.1. PU and CyanoCoating samples were disinfected, as described in Section 4.5.2., being then dried with argon stream in a flow hood and transferred to a 24-well tissue culture polystyrene plates (TCPS, Sarstedt, Nümbrecht, Germany). Then, 100 μL of inoculum (1.0 × 10^7^ CFUs/mL) were added to each well containing samples pre-hydrated in 900 μL of TSB for 30 min. After a 2 h incubation period at 37 °C, surfaces were rinsed three times with sterile PBS and re-incubated with 1000 μL of TSB during 24 h. After incubation, samples were rinsed five times with PBS to remove planktonic and loosely bound bacteria. Then, surfaces were transferred to 5 mL SARSTEDT tubes containing 1 mL of 0.5% Tween 80 in PBS and placed on ice, then sonicated using BactoSonicR (BANDELIN, Heinrichstraße, Berlin, Germany) at 160 W for 15 min, placed on ice for 5 min, sonicated again for 15 min and put on ice. As a control, the adjusted inoculum was submitted to the same sonication protocol to verify if the sonication applied interferes with microorganism viability. After, serial dilutions were done and plated for CFU counting. Results are the average of three replicates of three independent assays. 

To ensure that after sonication all bacteria were removed from the surfaces, PU and CyanoCoating samples were transferred to a 24-well plate and fixed with paraformaldehyde 4% (*v*/*v*) in PBS, for 30 min at RT. After rinsing with PBS three times, samples were stained with 4′,6-diamidino-2-phenylindole (DAPI) (0.1 μg/mL) for 30 min at RT, protected from light. Afterwards, samples were rinsed with PBS and transferred to an uncoated 24-well μ-plate (#82406, IBIDI) with the surface facing the bottom. The image acquisition and analysis were performed, described in Section 4.5.2. The adhesion reduction percentages and respective standard deviations were calculated, as described in Section 4.5.2. 

### 4.6. Encrustation Assay

The evaluation of the deposition of crystals on the surface of samples was performed using supplemented artificial urine medium, prepared as described by Cox and collaborators [26] (composition: Supplementary Table S3). Samples were immersed in 2 mL of AUS and incubated at 37 °C, 60 rpm for 7 days. These experiments were executed in triplicate. After 7 days, the samples were washed gently using distilled water to remove any salts that may be loosely deposited on the surface of the materials. Then, samples were dried in vacuum oven (Trade Raypa, Barcelona, Spain) overnight. The samples conductivity was enhanced by sputtering with Au/Pd for 60 s and 15 mA current using the SPI Module Sputter Coater equipment (Structure Probe, Inc., West Chester, PA, USA). The SEM / EDS analysis was performed using a High resolution (Schottky) Environmental Scanning Electron Microscope with X-Ray Microanalysis and Electron Backscattered Diffraction analysis (JEOL JSM 6301F / Oxford INCA Energy 350, Jeol, Peabody, MA, USA). Micrographs of the surfaces were taken using an electron beam intensity of 5 kV (accelerating voltage) and a magnification of 30×, at CEMUP (University of Porto, Porto, Portugal). 

### 4.7. Assessment of CyanoCoating Stability after Ethylene Oxide Sterilization 

#### 4.7.1. Water Contact Angle (WCA)

To assess the performance of CyanoCoating after clinically relevant sterilization procedure, samples were submitted to ethylene oxide (EO) sterilization (kindly performed at sterilization service of Hospital de São João, Porto, Portugal) and compared to samples disinfected with the protocol described in Section 4.5.2. (control samples). The ethylene oxide sterilization was performed using a sterilizer cabinet EOGas series 3 plus with ampoules system (Andersen Products, Essex, UK) during 16 h (4 h of sterilization plus 12 h of aeration) at 50 °C. 

Water contact angle measurements were performed using captive bubble method with a goniometer model OCA 15, equipped with a video CCD-camera and SCA 20 software (Data Physics, Filderstadt, Germany). Samples were tape glued to a microscope slide and placed with the surface facing the bottom in a quartz chamber filled with type I water. Subsequently, 10 μL bubbles of room air were introduced using a J-shaped syringe at a dose rate of 2 μL/s. Bubble profiles were fitted using tangent formula, to obtain the contact angle. Results are the average of two measurements of three replicates of three independent assays.

#### 4.7.2. Microbial Assays

In order to understand if EO sterilization process compromises bacterial adhesion in CyanoCoating surface, anti-adhesive assays performance was also evaluated, as described in Section 4.5 using *P. mirabilis* and *E. coli*.

### 4.8. Statistical Analysis

Statistical analysis was performed using Mann–Whitney test (*t*-test) and non-parametric Kruskal–Wallis test using the GraphPad Prism program version 6 (GraphPad Software, San Diego, CA, USA). Data is expressed as the mean ± standard deviation (SD) and p values of < 0.05 were considered significant.

## 5. Conclusions

Cyanobacteria are a prolific source of extracellular polymeric substances with particular characteristics that represent an untapped source of natural polymers for industrial applications, namely biomedicine. The evaluation of the cyanobacterial polymer-based CyanoCoating demonstrated that this coating is highly efficient in preventing the adhesion of most relevant uropathogens tested here, both in the presence of culture medium or artificial urine, when compared to medical grade PU. Moreover, a significant biofilm formation reduction was observed for three of these uropathogens, namely *E. coli*, *P. mirabilis,* and *C. albicans*. In addition, CyanoCoating is also promising on encrustation mitigation, and is rather stable after being subjected to an industrial sterilization technique (ethylene oxide). In the post-antibiotic era, strategies similar to the one reported here will play an important role as effective and non-cytotoxic solutions in the battle against CAUTIs.

## Supplementary Materials

The following are available online at https://www.mdpi.com/1660-3397/18/6/279/s1, Table S1: Metal contaminants assessment by Inductively Coupled Plasma–Atomic Emission Spectroscopy (ICP–AES). Table S2: Composition of the artificial urine medium according to Brooks et al., 1997 [32]. Table S3: Composition of the supplemented artificial urine medium according to Cox et al., 1987 [26]. Figure S1: Micrographs of *Proteus mirabilis*, *Klebsiella pneumoniae*, *Staphylococcus aureus* MRSA and *Candida albicans* cells adhered to polyurethane (PU) and CyanoCoating after 24 h incubation at 37 °C and stained with Draq5 and propidium iodide (PI) (scale bars—60 µm). Figure S2: Micrographs of *Escherichia coli*, *Proteus mirabilis*, *Klebsiella pneumoniae*, *Staphylococcus aureus* MRSA and *Candida albicans* cells adhered to polyurethane (PU) and CyanoCoating after 24 h incubation at 37 °C, in the presence of artificial urine medium (AUM), and stained with Draq5 and propidium iodide (PI) (scale bars—60 µm).
