# Supplementary material for: Natural Cyanobacterial Polymer-Based Coating as a Preventive Strategy to Avoid Catheter-Associated Urinary Tract Infections
Source: Mar Drugs. 2020 May 26;18(6):279. doi: 10.3390/md18060279 (PMC7344411; doi:10.3390/md18060279)
Supplement: Supplementary file 1 [file marinedrugs-18-00279-s001.pdf]

Supplementary material

Natural cyanobacterial polymer-based coating as a preventive strategy to avoid catheter-associated urinary tract infections

Bruna Costa[1,2], Rita Mota[1,3], Paula Tamagnini[1,3,4], M. Cristina L. Martins[1, 2,5], Fabíola Costa[1,2*]

[1]i3S – Instituto de Investigação e Inovação em Saúde, Universidade do Porto, Rua Alfredo Allen, 208, 4200-135 Porto, Portugal; bruna.costa@i3s.up.pt (B.C.); rita.mota@ibmc.up.pt (R.M.); pmtamagn@ibmc.up.pt (P.T.); cmartins@ineb.up.pt (M.C.L.M.); fabiolamoutinho@ineb.up.pt (F.C.)

[2]INEB – Instituto de Engenharia Biomédica, Universidade do Porto, Rua Alfredo Allen, 208, 4200-135 Porto, Portugal.

[3]IBMC – Instituto de Biologia Molecular e Celular, Universidade do Porto, Rua Alfredo Allen, 208, 4200-135 Porto, Portugal.

[4]Faculdade de Ciências, Departamento de Biologia, Universidade do Porto, Rua do Campo Alegre, Edifício FC4, 4169-007 Porto, Portugal.

[5]ICBAS – Instituto de Ciências Biomédicas Abel Salazar, Universidade do Porto, Rua Jorge de Viterbo Ferreira 228, 4050-313 Porto, Portugal.

Table S1: Assessment of metal contaminants by Inductively Coupled Plasma - Atomic Emission Spectroscopy (ICP-AES).

Sample	As (193.695 nm)	Cd (226.502 nm)	Hg (194.164 nm)	Pb (220.353 nm)
CyanoCoating (sample #1)	Nd	Nd	Nd	Nd
CyanoCoating (sample #2)	Nd	Nd	Nd	Nd
CyanoCoating (sample #3)	Nd	Nd	Nd	Nd
Limit of detection (µg/L)	5	1	1	10

Nd: non-detected

Table S2: Composition of artificial urine medium according to Brooks et al. [32].

Reagents	Amount g/L
Iron II sulphate heptahydrate	0.0012
Yeast extract	0.005
Uric acid	0.07
Lactic acid	0.1
Calcium chloride dihydrate	0.37
Citric acid	0.42
Magnesium sulphate heptahydrate	0.49
Creatinine	0.87
Potassium dihydrogen phosphate	0.95
Peptone L37	1.0
Di-potassium hydrogen phosphate	1.2
Ammonium chloride	1.3
Sodium bicarbonate	2.1
Sodium sulphate decahydrate	3.2
Sodium chloride	5.2
Urea	10.0

Table S3: Composition of supplemented artificial urine medium according to Cox et al. [26].

Reagents	Amount g/L
Urease	1.25
Magnesium chloride hexahydrate	3.0
Bovine albumin	5.32
Potassium dihydrogen orthophosphate	7.6
Urea	16.0
Calcium chloride hexahydrate	20.0

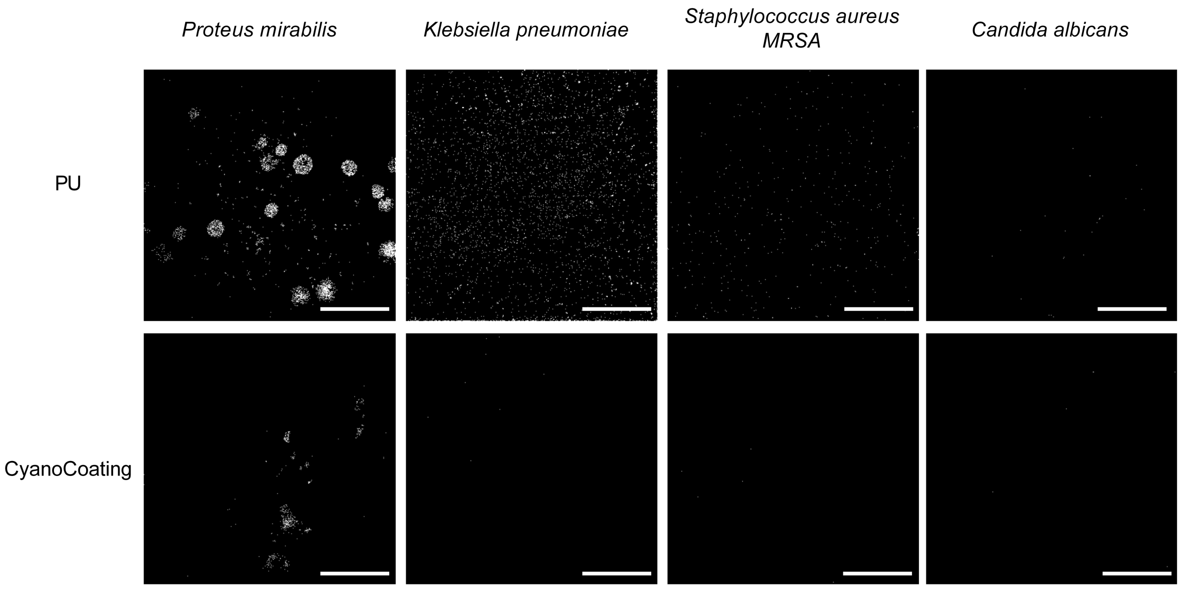

Figure S1: Micrographs of *Proteus mirabilis*, *Klebsiella pneumoniae*, *Staphylococcus aureus* MRSA and *Candida albicans* cells adhered to polyurethane (PU) and CyanoCoating after 24 h incubation at 37 °C and stained with Draq5 and propidium iodide (PI) (scale bars—60 µm).

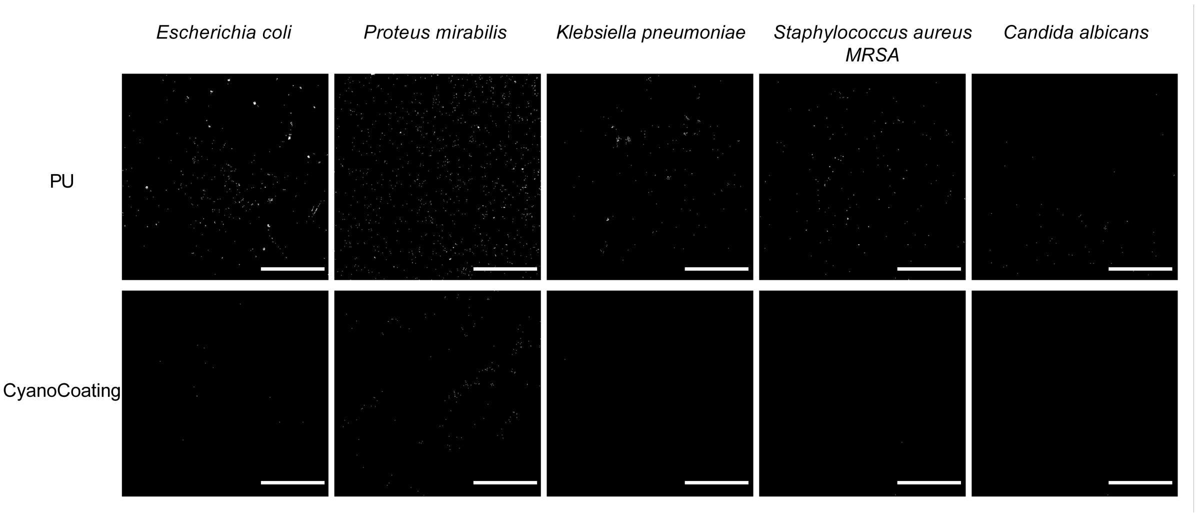

Figure S2: Micrographs of *Escherichia coli*, *Proteus mirabilis*, *Klebsiella pneumoniae*, *Staphylococcus aureus* MRSA and *Candida albicans* cells adhered to polyurethane (PU) and CyanoCoating after 24 h incubation at 37 °C, in the presence of artificial urine medium (AUM), and stained with Draq5 and propidium iodide (PI) (scale bars—60 µm).
